# How does government-backed finance affect SMEs’ crisis predictors?

**DOI:** 10.1007/s11187-023-00733-x

**Published:** 2023-02-27

**Authors:** Lorenzo Gai, Maria Cristina Arcuri, Federica Ielasi

**Affiliations:** 1 Department of Economics and Management, University of Florence, Via Delle Pandette, 9, 50127 Florence, Italy; 2 Department of Economics and Management, University of Parma, Via Kennedy, 6, 43125 Parma, Italy

**Keywords:** Business continuity, Central Guarantee Fund, Credit risk, Public guarantees, SMEs, G21, G28, G33

## Abstract

This paper estimates the impact of public guarantees on crisis predictive indicators among small and mid-size enterprises (SMEs). We use a confidential database provided by the Italian Ministry of Economic Development on the universe of guarantees granted by the Central Guarantee Fund. We apply difference-in-difference regressions and propensity-score matching estimators to a sample of approximately 40,000 SMEs over the 2010–2018 period. We find that obtaining a public guarantee improves profitability both in the short- and medium-term. On the other hand, SMEs’ financial health worsens in the short run, but financial burdens are alleviated 2 years after the issuance of a guarantee. The economic and financial effects of government-backed loans are amplified for micro-sized firms, companies operating in the service sector and direct guarantees. Our results can thus support public authorities in designing credit guarantee schemes capable of preventing SMEs’ zombification and protecting them from the risk of debt overhang.

## Abstract (plain language summary)

Access to public credit guarantee schemes negatively impact SMEs’ financial equilibrium, but their recovery occurs 2 years after guarantee issue. How does one select eligible firms to prevent zombification? Using a confidential dataset provided by the Italian Ministry of Economic Development on guarantees issued by the Central Guarantee Fund, we investigate this topic with an unprecedented level of salience. Our findings reveal the need for cautious interventions on firms in financial distress and for the introduction of stress tests to select beneficiaries. Our results show that specific guarantee lines could be applied for direct guarantees granted to micro-sized enterprises and companies operating in the service sector to maximize the additionality of public resources. This study has practical, policy and societal implications, guiding SMEs in their assessment of the overall medium-term effects of guarantees and policy-makers in their rethinking of guarantee schemes to resolve trade-offs between effectiveness and sustainability.

## Introduction

Throughout European countries, plans for recovery and resilience following the coronavirus disease (COVID-19) pandemic have included the strengthening of public guarantee programmes. In addition to moratoria on loan repayments, tax deferrals, grants, equity facilities and wage subsidies, public guarantee schemes represent a fundamental instrument within the countercyclical public policy toolkit deployed by governments to support the liquidity needs of firms, particularly small and mid-size enterprises (SMEs) (Anderson et al., 2021; Lehmann & Lenaerts, 2021).

The large increase in the outstanding guarantee volume, as well as the number of supported firms, raises important questions on both the sustainability of public finances and the actual effectiveness of these programmes in terms of economic growth (Ciani et al., 2020; European Banking Authority, 2020).

This study aims to evaluate the effects of government-backed finance on SMEs’ crisis indicators 1 year and 2 years after guarantees are issued. We attempt to understand whether public guarantees are able to impact the business continuity prospects of guaranteed SMEs by comparing them to non-guaranteed SMEs. We specifically address the following research questions:

1. What is the impact of a public guarantee on the warning signs of a business crisis?
2. Does the impact of public guarantees on the warning signs of a business crisis vary according to the characteristics of the eligible guaranteed SMEs?
3. Does the impact of public guarantees on the warning signs of a business crisis vary according to the technical form of the issued guarantee (i.e. the type of financial institutions involved in the process)?

To answer these questions, we use a confidential database provided by the Italian Ministry of Economic Development that includes all guarantees issued by the Italian Central Guarantee Fund (CGF) from 2012 to 2016, which we integrate with data downloaded from the Aida Bureau van Dijk database for a period ending in 2018. We use difference-in-difference regressions (DiD or diff-in-diff) and propensity-score matching estimators to compare a proprietary sample of 17,810 guaranteed firms with a sample of approximately 21,000 comparable SMEs.

Papers that analyse the impacts of government-sponsored credits typically focus on a specific country given the need to compare transactions regulated by the same legislation (Beck et al., 2010; Decramer & Vanormelingen, 2016). In particular, this paper focuses on the Italian public guarantee fund. Italy is an important case for this topic at an international level for different reasons. First, credit guarantee schemes traditionally play a crucial role in easing SMEs’ access to finance in Italy, and in recent years, guarantee programmes have played an increasing role in this country (Caselli et al., 2019; Ciani, et al., 2020; Lagazio et al., 2021). Government-guaranteed loans account for approximately a 12% share of SME loan stock and 8% of the GDP, representing one of the highest such rates in Europe (OECD, 2020).

According to Anderson et al. (2021), the current design choices of the credit-support programmes in Italy have resulted in risks related to public finance and SME zombification^1^ that are higher than those observed in France, Germany, Spain, and the UK. An in-depth analysis of SMEs’ selection criteria and effects on their economic and financial equilibrium is relevant at an international level to rethink guarantee programmes during a recovery phase and limit public funds at risk. This study could support policymakers in revising the design choices applied to the CGF to better manage the trade-offs between the programme’s reach and its risks.

This study makes a multifaceted contribution to the existing literature concerning the economic and financial additionality of public guarantee schemes. Concerning the economic additionality, the literature investigates the benefits provided by guarantees for firms’ profitability, investments and employment (Bertoni et al., 2018; Caselli et al., 2019; Heshmati, 2013; Kang & Heshmati, 2008). Furthermore, regarding financial additionality, the literature verifies the impacts of public guarantees on the financial conditions of supported loans by analysing the effects on interest rates, collateral, loans’ amount and maturity (Bartoli et al., 2013; Calcagnini et al., 2014; Ciani et al., 2020; Columba et al., 2009; Cowan et al., 2015; Zecchini & Ventura, 2009). This study intends to merge these two perspectives by verifying how obtaining public guarantees can contribute to both the economic and financial equilibrium of secured companies from a broader perspective of business continuity.

To achieve this goal, the impact of public guarantees is investigated by verifying the effects on a package of indicators introduced by the Italian legislator as a part of the harmonization of insolvency and restructuring law in the European Union (EU) to obtain predictive signals of corporate crises. Public guarantee funds pursue economic policy objectives but must simultaneously ensure sustainability in the use of public funds for the benefit of the taxpayers. Therefore, the selection of eligible companies while considering business continuity prospects over time from a forward-looking perspective is essential. Then, the study measures the impacts of new guaranteed loans on the overall health of the company as measured by predictive indicators of corporate crises. The overall package of these indicators was not previously investigated by the international literature and represents one of the first empirical tests applying the recent Code of the Business Crisis and Insolvency (Legislative Decree 14/2019,—hereinafter, the Code). The Code aims to create functional instruments to facilitate the early identification of the debtor’s financial crisis to prevent insolvency and, when such efforts fail, manage the insolvency with the aim of overcoming the crisis and restoring the company to profitability. Therefore, the warning signals to prevent a crisis investigated in this study include not only economic and capital adequacy indicators but also debt sustainability indices, which are particularly relevant in evaluating the effectiveness of public support for access to new financing. Regarding this issue, the literature has paid limited attention to the impact of public programmes on firms’ liquidity and the overall structure of corporate debts. We attempt to contribute to filling this gap by studying the impact of guarantees on the overall cost of corporate debt and debt sustainability with cash flows.

The effects of public guarantees on SMEs’ going concern were investigated in the context of economic and financial crises (Bartoli et al., 2013; Briozzo & Cardone-Riportella, 2016; Oh et al., 2009) and amid the COVID-19 crisis (AECM, 2021; European Bank for Reconstruction and Development, 2020). We aim to contribute to the literature concerning this topic by focusing on an updated and statistically relevant sample covering a recovery period after a crisis. This is extremely relevant today in relation to future decisions about guarantee programmes after the COVID-19 pandemic emergency. We analyse all the guarantees issued from 2012 to 2016, namely after the international financial crisis and the 2011 European sovereign debt crisis. We study the effects of these guarantees on firms’ performance and business continuity prospects until 2018, before the new crisis related to the COVID-19 pandemic emerged. Compared to studies focusing on the peaks of crises, a more neutral period from negative economic conditions is, therefore, investigated. Since the impacts of programmes designed to support access to finance vary across the business cycle, this contribution is particularly relevant (Jorion & Zhang, 2009).

Moreover, to the best of our knowledge, few papers conduct a deep analysis of the role of public guarantees by considering the type of issued guarantee and the related financial institutions that intervene in the process (Bartoli et al., 2013; Cardone-Riportella et al., 2013; Caselli et al., 2021; Columba et al., 2010). We complement the previous findings in this field by verifying the role of different players, other than the focal public guarantee fund and the beneficiary firm involved in the guarantee granting process according to the specific form of public intervention. In this study, we determine whether the intervention of a Mutual Guarantee Institution (MGI) that is able to cover a loan before the enforcement of the public guarantee contributes to mitigating the effects we investigated.

We find that obtaining a public guarantee generally improves the business continuity prospects of SMEs in the medium-term, but firms’ economic and financial performance are affected in different ways. In general, the issuance of a public guarantee contributes to increasing the differences between guaranteed and non-guaranteed firms. However, the guarantees initially impose opposing pressures on SMEs’ economic and financial conditions: an increase in profitability is accompanied by a deterioration of the SMEs’ financial structure and conditions. However, while the trend in profitability remains positive and continues to grow even 2 years after a guarantee is granted, its negative impact on the level of indebtedness and related costs is absorbed in the medium-term.

Our results confirm that the magnitude of the aforementioned effects varies according to the size and industry of the guaranteed SME and on the type of intervention employed by the CGF. The most evident impacts are observed among microenterprises and companies operating in the service sector. When an MGI intervenes, more moderate effects on companies’ profitability are produced, but the negative effects on firms’ financial structures are decidedly more contained, even in the short-term.

This paper is structured as follows. Section 2 describes the main characteristics of the Italian CGF. Section 3 reviews the literature on the economic and financial additionality of public guarantee schemes. Section 4 describes the study’s data and methodology. Section 5 analyses the main results, and Section 6 concludes by highlighting some interesting implications for the design and implementation of public guarantee schemes for SMEs during recovery times.

## Public guarantee schemes and the Italian Central Guarantee Fund

In the recent past, many governments introduced or expanded public guarantee schemes by increasing the total volume of guarantee funds, the guarantee volume per eligible company, the coverage rate of loans (the maximum percentage of a loan that can be covered by a guarantee) and the repayment periods of guaranteed loans and reducing guarantee fees and documentation requirements (AECM, 2021; EBRD, 2020).

Public guarantee programmes are among the main tools used to support access to finance during and after a recession period. As a strategic response to a financial and economic crisis, these schemes seem to be more effective than other direct public interventions (Arping et al. 2010; OECD, 2011). Moreover, they are a form of market-friendly intervention that leaves the final decisions on loans to private parties (Beck et al., 2010; OECD, 2013). Finally, most supported credit is supposed to be repaid, and public funds are typically used only for a fraction of the stated amounts.

The Italian CGF was established by Law No. 662/96 and has been active since 2000. The CGF specifically targets micro-enterprises and SMEs as defined by the European Commission in the EU Recommendation 2003/361.^2^ Companies operating in any economic sector can be guaranteed, except for financial corporations.

The CGF is funded by the Italian State and the European Union. In addition to European structural funds, the scheme receives counterguarantees from the European Investment Fund. The CGF is managed on the behalf of the Ministry for Economic Development by Mediocredito Centrale; this bank has been fully owned by the Italian government since 2017 and is directly supervised by the Bank of Italy under the oversight of the European Central Bank. The fund provides first demand guarantees that are subject to capital relief for banks. It is possible to combine the interventions of the CGF with a guarantee issued by an MGI, to achieve 100% coverage of a loan. In particular, a guarantee can be granted by the public scheme in the following ways: (1) a direct guarantee can be activated to cover a loan granted by a bank, a leasing company or other financial intermediaries without directly intervening in the lender-borrower relationship; or (2) a counterguarantee^3^ or a reinsurance^4^ can be given to an MGI, which acts as a first-level guarantor. Then, the role of the CGF is facilitating SMEs’ access to credit by issuing public guarantees that are complementary or in lieu of private or mutual guarantees. For this reason, it is mainly addressed to SMEs and innovative start-ups, which are often disadvantaged in terms of higher cost of credit or worst covenants. Despite the purpose of economic development and the need to simplify access to the tool, it is necessary to avoid procedures able to benefit less deserving businesses. The models implemented by the public guarantor for evaluating and selecting eligible companies should optimize the allocation of public funds for a greater impact on the economic system, the prevention of over-indebtedness, the control of risks faced by the government as a last resort lender and the limitation of moral hazard behaviours of banks and mutual guarantee institutions. The regulation of the CGF defines the content of the scoring model used for the selection of eligible firms.^5^ During the focal period, the evaluation of creditworthiness for guarantee eligibility is carried out by calculating a predictive measure of SMEs’ capital, economic and financial risk profile, which defines the maximum probability for default of the potential beneficiary. This assessment is carried out on the last two financial statements approved by the company on the date of submission of the request for admission to the CGF. The model applies different algorithms based on the following characteristics of the SME: legal form, accounting regime (ordinary or simplified regime) and economic sector. The algorithms take into consideration different balance sheet ratios, selected according to their significance in assessing the creditworthiness of the final beneficiaries. For example, the financial–economic evaluation module for limited liability companies in an ordinary accounting regime operating in the trade and service sectors involves an algorithm that includes the following balance sheet indicators: current ratio (current assets/current liabilities), capitalization (equity capital over total liabilities), interests coverage ratio (EBITDA over financial interests) and incidence of core operating profits on sales (EBITDA over sales).

SMEs that exceed a predefined probability of default cannot access the Fund. Eligible firms, on the other hand, have different conditions for accessing the Fund based on their obtained score. For example, national regulations establish the maximum coverage percentages of the guarantee to the total amount of the loan and the maximum guaranteed amount, which can vary based on the obtained score. The guarantee never covers 100% of the loan, but the coverage rate can reach 80% of the loan for direct guarantees and 90% for a counter-guarantee to an MGI whose guarantee cannot, however, exceed 80% of the loan. Such conditions also differ according to the amount and type of operation guaranteed, in the short- or medium–long-term, for liquidity or investments.

During and after relevant economic or financial shocks, when firms’ survival is exposed to growing risks, the CGF has experienced significantly increasing rates in both the applications for admission received and accepted. In particular, the role of the CGF was strengthened during the international financial crisis started in 2008 and after the European sovereign debt crisis started in 2010. It was refinanced with approximately two billion euros between 2008 and 2012 and then again between 2013 and 2014 with 1.2 billion euros. Afterwards, during the COVID-19 pandemic, the Italian government adopted extraordinary measures that reinforced and significantly expanded the programme by broadening its coverage, relaxing its eligibility criteria and increasing its endowment.

During the period covered in this study, the number of requests forwarded to the CGF increased by approximately 60%, the number of requests accepted has risen by almost 70%, while the number of companies guaranteed grew by approximately 60%.^6^ From the statistics provided by the CGF, the most significant growth featured medium- and long-term operations, confirming the fact that the instrument of the public guarantees was transformed over time from an exceptional financial intervention into a tool for supporting investments and corporate growth.

During the COVID-19 emergency, the functioning of the CGF has been temporarily modified. Currently, it could be necessary to rethink the structure of the programme during the post-pandemic era.

## Literature review and hypotheses

This paper is related to the literature that studies the effects of public interventions in terms of supporting the access to finance of micro-sized enterprises and SMEs. This focus on smaller companies is particularly relevant since such companies are a crucial element of most European countries: in 2020, they accounted for more than 99.8% of all the businesses in the EU-27 non-financial business sector, contributed more than half of the value added of the Union and accounted for approximately two thirds of the total employment of the private sector with over 90 million employees (European Commission, 2021). Micro-sized enterprises and SMEs represent an essential source of entrepreneurship and innovation for European economic growth and sustainability, and their survival is essential for national economies across the EU (European Commission, 2020; European Parliament, 2021).

The recovery and development of SMEs are currently a priority for policymakers, which has been confirmed by the recent updates of the Small Business Act, the role assumed by SMEs in the Next Generation EU plan and the publication of the SME strategy for a sustainable and digital Europe (European Commission, 2020).

The contribution of SMEs to economic and social development can be hampered by difficulties related to accessing credit (Abraham & Schmukler, 2017; Stiglitz & Weiss, 1981). SMEs rely on bank loans more heavily than larger firms as they have limited access to alternative funding sources (Abraham & Schmukler, 2017; Fazzari et al., 1988). In the context of access to banks, SMEs have traditionally faced tougher credit conditions than larger companies for different reasons, including information asymmetry and high-risk perceptions (Berger & Udell, 2006; Cowling, 2010), high administrative costs for small-scale lending (Cowling & Mitchell, 2003; Rostamkalaei & Freel, 2016) and a lack of collateral (Beck et al., 2008; Jaffee & Russell, 1976; Stiglitz & Weiss, 1981). Moreover, SMEs tend to be more vulnerable to shocks since they are less diversified in their sources of revenue. Thus, credit constraints affect SMEs strongly, and this effect is more pronounced during times of crisis (Albareto & Finaldi Russo, 2012; Beck et al., 2006; Casey & O’Toole, 2014).

Regarding credit support instruments, loan guarantee funds have been among the preferred programmes in the largest European economies, where they are used as a supplementary tool for supporting viable and creditworthy companies that are illiquid and funding-constrained (Anderson et al., 2021).

As analysed below, the existing literature studies the effects of public guarantee programs for SMEs in terms of “additionality” with reference to the following two dimensions: (1) economic additionality and (2) financial additionality.

Economic additionality refers to the improved economic performance that beneficiary companies experience due to increased access to finance (Leone & Vento, 2012; Levitsky, 1997). Researchers measure the impact of public guarantees in terms of the following:

1. Employment by considering increases in the number of employees or the employment growth rate (Armstrong et al., 2010; Riding et al., 2007);
2. Sales and profitability in terms of ROE and ROI (Asdrubali & Signore, 2015; Bertoni et al., 2018; Caselli et al., 2019; Kang & Heshmati, 2008; Mole et al., 2009); and
3. Investments in terms of both working capital and investment capital (Ono et al., 2010).

Some studies show the importance of economic additionality for policymakers since the growth of guaranteed firms can contribute to improving gross domestic product (Kang & Heshmati, 2008; Panetta, 2012) and tax revenues (Boocock & Shariff, 2005; Schmidt & van Elkan, 2010).

However, other studies do not confirm the positive effect of guarantees on firms’ performance (Gozzi & Schmukler, 2015; Samujh et al., 2012; Uesugi et al., 2010), and some show that negative effects in terms of probability of default can emerge (Lelarge et al., 2010). In contrast to collateral provided directly by a firm, a public guarantee can determine the occurrence of moral hazard behaviours and depress the efforts of both companies and banks in mitigating risks (Myers & Majluf, 1985).

The actual effectiveness of a guarantee programme can depend on the selection criteria used and the level of opacity of eligible firms, which is affected by the size of the company and its sector (Graham, 2004; Uesugi et al., 2010). Martìn-Garcìa and Morán Santor (2021) show that public credit guarantees have the greatest impact on micro-enterprises (those with fewer than 10 employees), which are opaquer and, consequently, more affected by barriers to finance. Economic additionality also depends on the industry in which a firm operates. Each sector is characterized by different levels of growth opportunities and investment needs (Myers, 1984), and the intensity of expected default rates strongly varies across industries (EBRD, 2020). Moreover, a firm’s sector affects the level of opacity by impacting the rate of asset tangibility (Van der Wijst & Thurik, 1993). As a consequence, a firm’s sector affects its potential for public credit support interventions. The more carefully firms are selected in terms of size and sector, the better the impact on the economic system (Davidsson et al., 2008; Samujh et al., 2012).

Hence, we posit the following hypotheses:

- H1: Public guarantees have positive effects on SMEs’ economic performance during an economic recovery period.
- H2: The effects of public guarantees on SMEs’ economic performance during an economic recovery period are conditioned by the size of companies.
- H3: The effects of public guarantees on SMEs’ economic performance during an economic recovery period are conditioned by the economic sector in which the companies operate.

Financial additionality refers to the financial impact of public guarantee programmes on credit access conditions and measures the following effects: increases in the resources allocated by the banking system to guaranteed companies in terms of the size of loans or extensions of credit maturities and reductions in the interest rates, transaction costs and collateral applied to loans (Riding et al., 2007;).

The existing literature confirms that public guarantee schemes increase the amount of credit available to guaranteed firms (Cardone-Riportella et al., 2013; Cowan et al., 2015; Ughetto et al., 2017; Zecchini & Ventura, 2009) and enhance their financing conditions (Calcagnini et al., 2014; Columba et al., 2010; Cowling, 2010; Gozzi & Schmukler, 2015; Vogel & Adams, 1997), emphasizing the positive impact of these programmes on guaranteed loans. These effects are emphasized following the introduction of the Temporary Framework for State aid measures by the European Commission in March 2020. According to this framework, financial institutions must pass the advantages of public guarantees to final beneficiaries in the form of guaranteed loan volumes that are higher or interest rates that are lower than those that would have prevailed without public intervention (European Investment Bank, 2021).

The literature concerning financial additionality studied the impact of public interventions on the financial conditions of specific guaranteed loans (Calcagnini et al., 2014; Cardone-Riportella et al., 2013; Columba et al., 2010; Cowan et al., 2015; Cowling, 2010; Gozzi & Schmukler, 2015; Riding et al., 2007; Ughetto et al., 2017; Zecchini & Ventura, 2009). However, the effect of such guarantees on a firm’s overall financial equilibrium is less investigated, and this effect could impact its very survival (Caselli et al., 2021). It seems that public guarantees can be associated with higher levels of default among guaranteed firms and an increase in the long-term resources available to SMEs, which could alter their financial structures (Abraham & Schmukler, 2017; Lelarge et al., 2010). Moreover, a guarantee can encourage moral hazard behaviour by the subsidized firm, which could worsen the company’s risks and creditworthiness. In this way, public guarantees can impact the overall costs incurred by companies against all their debt (Gai et al., 2016; Lagazio et al., 2021). To contribute to this strand of the literature, we sought to verify the impact of guarantees on the overall level of indebtedness and the general credit access conditions of guaranteed companies while comparing them to nonguaranteed companies. We posit the following hypothesis:

- H4: Public guarantees have negative effects on SMEs’ financial structure and financial costs.

The behaviours of selected companies and the impact of the guarantee on their financial equilibrium can be conditioned by the number and nature of the financial institutions that intervene in the loan process and the evaluation of credit risk. Existing literature shows that the intervention of a guarantor who has information advantages over the lender can serve to mitigate asymmetric information problems (Caselli et al., 2021; Gai et al., 2016). This should be true for MGIs that are particularly rooted in the focal territory and that have access to soft and nonfinancial information, both in the precontractual phase and in the context of monitoring borrowers (Bartoli et al., 2013; Cardone-Riportella et al., 2013; Caselli et al., 2021; Columba et al., 2010). The peer screening and peer monitoring carried out by the members of MGIs can enhance the reputation of companies, which may counter the potential increase in general financial conditions due to so-called signalling effects (Bartoli et al., 2013; Columba et al., 2010). Nevertheless, the intervention of an MGI as a first-level guarantor and the use of the public programme as a counterguarantee can increase the overall coverage ratio. This reduction in the portion of credit risk retained by banks may induce relaxed loan selection processes and encourage adverse selection (Saito & Tsuruta, 2014). Moreover, firms must pay a specific fee for the intervention of an MGI, and this can produce adverse selection effects if riskier companies are willing to face the costs of both a guarantee and a counterguarantee (Columba et al., 2009). This may lead to an increase in demand for guarantees through MGIs for nonviable borrowers (Kuniyoshi & Tsuruta, 2014). The overall effect of the inclusion of an MGI other than the lending bank is uncertain. Therefore, we posit the following hypothesis:

- H5: The effect of public guarantees on SMEs’ financial structure and financial costs is conditioned by the technical form of intervention and the type of intermediary involved in the process.

## Data, sample and methodology

### Data and sample

Our empirical analysis is based on the universe of guarantees granted by the Italian CGF from 2012 to 2016. The data are related to 17,810 SMEs and are sourced from a confidential database provided by the Italian Ministry of Economic Development. The granularity of the available data facilitates a refined analysis of the effectiveness and impact of the CGF. For each guarantee issued, we have the following information: the date and amount of the guarantee, type of intervention (guarantee or counterguarantee), the accessed loan and the sector and geographical area of the guaranteed firm. In the dataset, we include the balance sheet information of the guaranteed firms, which is sourced from the Aida Bureau van Dijk database. To cover the periods before and after each guarantee was granted, we collect data from 2010 to 2018. The values are downloaded from the 2 years before the issuance of each guarantee for bias correction; financial and economic data from 2017 and 2018 are used to measure the impacts of the public guarantees. Table 1 shows the main characteristics of our sample.

**Table 1:** Characteristics of guarantees issued by the CGF during the 2012–2016 period

	2012	2013	2014	2015	2016	All period
Average loan amount (€)	253,231	231,833	213,608	208,705	219,207	208,704
Loan coverage ratio (guarantee/loan) (%)	57.87	58.83	61.19	62.29	60.93	62.29
Direct guarantee (%)	52.31	49.84	52.03	58.54	59.04	54.31
Counter guarantee (%)	47.69	50.16	47.97	41.46	40.96	45.69

We develop a consistent control sample of comparable firms that did not receive public guarantees during the same period (Rubin, 2004). We download the entire record of Italian companies from the Aida Bureau van Dijk database, and we identify 20,988 firms that are comparable to the guaranteed firms in terms of size, geographical area and industry. We exclude any companies that received guarantees from the CGF from the control sample.

To identify the determinants of access to the CGF, we refer to variables used in the literature to evaluate firms’ creditworthiness (Altman, 1968; Beaver, 1966; Lin et al., 2011; Min et al., 2006; Ohlson, 1980). According to the Asset Quality Review provisions elaborated by the European Central Bank and updated on 20 June 2018, to assess the creditworthiness of SMEs, banks must include in their analyses a set of indicators related to firms’ profitability, financial structure, solvency and liquidity. In this regard, the Italian CGF adopts specific financial indicators to define the eligibility of individual companies to obtain a public guarantee. Consistent with the eligibility criteria of the CGF, we compare the two samples of guaranteed and unsecured companies while considering the following firm characteristics: ROE and ROS for profitability, debt-to-equity ratio for financial structure, interest coverage ratio for solvency and current ratio for liquidity.

Previous studies (Berger & Udell, 1998; De Jong et al, 2008; Denis & Mihov, 2003; Leland & Pyle, 1977; Rajan & Zingales, 1995) show that the following additional variables should be included in analyses comparing firms’ ease of access to finance: size (measured by the log of total assets and the number of employees), geographical area (measured by dummy variables), economic sector (measured by dummy variables) and age (in number of years). Table 2 reports the descriptive statistics of the two subsamples with reference to 2011 that is the year before the period under analysis.

**Table 2:** Descriptive statistics of the sample (2011)

		Guaranteed SMEs		Control sample	
	Description	Mean (a)	Standard deviation	Mean (b)	Standard deviation
Firm size (percent)					
Micro	SMEs with a n. of employees less than or equal to 10	48.55	0.004	48.54	0.003
Small	SMEs with a n. of employees between 11 and 50	41.08	0.003	37.64	0.002
Medium	SMEs with a n. of employees between 51 and 250	10.51	0.003	13.19	0.002
Geographical area (percent)					
North	SMEs located in Northern Italy	53.31	0.004	62.41	0.003
Central	SMEs located in Central Italy	18.96	0.003	13.20	0.002
South and Islands	SMEs located in Southern Italy or on islands	27.67	0.002	24.38	0.001
Economic sector (percent)					
Agriculture	SMEs in the agriculture industry	5.98	0.002	6.85	0.002
Manufacturing	SMEs in the manufacturing industry	1.52	0.004	1.66	0.003
Services	SMEs in the service industry	91.26	0.003	89.87	0.003
Trade	SMEs in the trade industry	1.24	0.003	1.62	0.002
Other firm characteristics					
Firm age	N. of years	13.45	0.080	20.57	0.107
Total assets	Log of total assets	7.53	0.0132	9.72	0.0131
Financial data					
ROE (return on equity)	Net profit/equity	9.29	0.223	8.92	0.261
ROS (return on sales)	EBITDA (earnings before interest, taxes, depreciation and amortization)/sales	7.49	0.175	6.95	0.231
Interest coverage ratio	EBIT (earnings before interest and taxes)/financial interest	11.93	0.278	26.80	0.470
Debt-to-equity ratio	Total liabilities/equity	3.19	0.310	1.94	0.382
Current ratio	Short-term assets/short-term liabilities	0.91	0.005	1.13	0.009

Source: CGF and Aida Bureau van Dijk. The values shown in the table refer to the year 2011

The guaranteed SMEs have higher profitability than the non-guaranteed SMEs. Moreover, they are younger and smaller. The guaranteed firms have higher debt-to-equity ratios and lower solvency as measured by the interest coverage ratio. Finally, the guaranteed SMEs have a lower liquidity ratio than the firms in the control sample.

### Methodology

Several techniques can be applied for the evaluation of socioeconomic programmes, such as regression-adjustment, matching, diff-in-diff, instrumental variables and regression discontinuity designs (Cerulli, 2015). In particular, several authors (Autio & Rannikko, 2016; Bryson et al., 2002; Lechner, 2002; Marino et al., 2016; Oh et al., 2009) analyse the effects of public policy using matching methods. According to Zecchini & Ventura (2009), most existing studies investigating the effects of guarantees utilize linear (Beck et al., 2010) and nonlinear (Columba et al., 2010) regression methods and impact evaluation techniques (Briozzo & Cardone-Riportella, 2016). In this study, we use propensity score matching and diff-in-diff.

To address potential endogeneity and the selectivity problem of the evaluation of public programmes, first, we evaluate the effects of public guarantees on business continuity by adopting propensity score matching methodology. We verify their impact on both profitability (measured by the return on investment — ROI) and SMEs’ ongoing concern indicators (measured by crisis indicators). To evaluate economic additionality, we choose the ROI because it can be used to measure, in terms of profitability, the effect of new investments financed by guaranteed loans (Uesugi et al., 2010). Furthermore, for the analysis of business continuity prospects with a financial perspective, certain crisis indicators are identified in the mentioned Business Crisis and Insolvency Code. The Code, whose entry into force was postponed by the PNRR 2 decree to 15 July 2022, aims to safeguard business continuity and protect employment. The Code sets up early warning procedures, according to which entrepreneurs and administrative and control entities must conduct careful analyses to detect the first symptoms of a crisis and subsequently take timely action to prevent insolvency. The main business crisis indicators identified by the Code and used in this study are the following: financial interest/sales, debt/equity, short-term assets/short-term liabilities, cash flow/total assets and (tax debts + social security debts)/total assets.

We use a matching estimator, ${Y}_{1i}$, that is the value of the outcome variable (i.e. the change in profitability growth and crisis indicators, calculated as the difference between the focal value after the first and the second year following the issuance of the guarantee and the value at the time of the issue), where ${unit}_{i}$ is subject to treatment (i.e. the granting of a guarantee is the “treatment variable”) and ${Y}_{0i}$ is the value of the same variable prior to treatment. The effect of the public guarantee granted to firm *i* is $e_i=\;Y_{1i}\;-\;Y_{0i}$ that is the variation in the mentioned indicators after the guarantee is granted. The sample of guaranteed firms is the “treated” group. The average treatment effect (ATT) refers to the average difference that it is observed if every firm in the treated group receives treatment instead of none of them receiving treatment. The formula is as follows:

1 $$\mathrm{ATT}\;=\mathrm E\;\left({\mathrm Y}_{1\mathrm i}\left|{\mathrm T}_i=1\right.\right)-\mathrm E\left({\mathrm Y}_{0\mathrm i}\left|{\mathrm T}_i=1\right.\right)$$

where T = 1 if firm *i* obtained a guarantee and T = 0 if it did not. E(${\mathrm{Y}}_{0\mathrm{i}}|{T}_{i}=1)$ is not directly observed, and matching estimators allow us to assign the missing potential outcomes ${\mathrm{Y}}_{0\mathrm{i}}$ to the treated firms using those of comparable untreated firms. We apply propensity score matching with the K-nearest neighbour algorithm (Li, 2012). The ATT is computed by selecting *n* comparison units with the closest propensity scores to those of the treated unit to be analysed. Since the relevance of the variables depends on the extent to which they affect the probability of treatment (the granting of a CGF guarantee), we choose the covariates by conducting a probit regression. The formula is as follows:

2 $$\begin{array}{c}\mathrm{Pr}\left(\mathrm{CGF}=1\left|{\mathrm{X}}_{\mathrm{i},\mathrm{t}-1}\right.\right)=\upphi \left({\upbeta }_{0}+{\upbeta }_{1}{\mathrm{ROE}}_{\mathrm{t}-1}+{\upbeta }_{2}{\mathrm{ROS}}_{\mathrm{t}-1}+{\upbeta }_{3}\mathrm{Debt}/{\mathrm{Equity}}_{\mathrm{t}-1}\right)+\\ {\upbeta }_{4}\mathrm{ Current\ }{\mathrm{ratio}}_{\mathrm{t}-1}+{\upbeta }_{5}\mathrm{Interest\ coverage }{\mathrm{ratio}}_{\mathrm{t}-1}+{\upbeta }_{6}\mathrm{Log\ Total\ Assets}_{\mathrm{t}-1}\\ {\upbeta }_{7}\mathrm{Geographical\ area}+{\upbeta }_{8}\mathrm{ Sector}+{\upbeta }_{9}\mathrm{Age}+{\upbeta }_{10}\mathrm{Year}\end{array}$$

The propensity scores represent the conditional probability of participating in the CGF guarantee programme given the values of the variables included in the analysis. The dependent variable is a binary variable that assumes a value of 1 if the focal firm participates in the CGF programme and 0 otherwise. ${\mathrm{X}}_{\mathrm{i},\mathrm{ t}-1}$ is the vector of independent variables relative to firm *i* observed in the year before the guarantee is granted (*t-1*). We use the k-nearest matching algorithm and identify k-matched (control) observations from the sample of non-guaranteed firms, namely the “untreated” firms, for each observation. The control observations are the non-guaranteed firms that are closest to the treated observations in terms of propensity score. The number of control observations, *k*, is arbitrarily determined. We estimate the average treatment of the sample with four equally weighted matches, controlling for heteroskedasticity (Abadie et al., 2001). Indeed, Abadie & Imbens (2002) demonstrate that four matches can be used to reduce the mean-squared error. Thus, we estimate the treatment effect of the CGF by matching each treated observation in year *t* with the four nearest untreated observations in the same calendar year. The distance is measured in terms of propensity scores.

To answer our research questions, we run the matching estimator on the whole sample and on the subsamples based on the size (micro, small or medium) and industry (service, agriculture, commerce or manufacturing) of each firm. We proxy the size of each firm based on its number of employees: micro-sized enterprises have up to 10 employees; small firms have between 11 and 50 employees; and medium firms have between 51 and 250 employees. Using the aforementioned k-nearest matching estimator, for each subsample, we estimate the effect of guarantees on the changes in profitability and the indicators of business crisis 1 and 2 years after the issuance of the guarantees. Then, we apply propensity score matching to each subsample.

To overcome the potential limitations of propensity score matching, we also apply the DiD methodology. Equation (3) represents our model:

3 $${\mathrm{Y}}_{\mathrm{i},\left(\mathrm{t}+\mathrm{n}\right)}={\upbeta }_{0}+{\upbeta }_{1}{\mathrm{dCGF}}_{\mathrm{i}}+{\updelta }_{0*}{\mathrm{d}}_{1}+{\updelta }_{2}{\mathrm{CGF}}_{\mathrm{i}*}+{\upbeta }_{*}^{\mathrm{^{\prime}}}{\mathrm{X}}_{\mathrm{i},\mathrm{t}-1}+{\uprho }_{\mathrm{t}}+{\mathrm{\alpha }}_{\mathrm{i}}+{\upvarepsilon }_{\mathrm{it}}$$

where Y is the outcome measure that is the ROI growth rate and the changes in the business crisis indicators (i.e. financial interest/sales, debt/equity, short-term assets/short-term liabilities, cash flow/total assets and (tax debts + social security debts)/total assets) of firm *i* at time *t*; *n* is equal to 1 and 2; ${\mathrm{dCGF}}_{\mathrm{i}}$ is a dummy variable equal to 1 for guaranteed firms and 0 otherwise; ${\mathrm{d}}_{1}$ is a dummy variable equal to 1 for the period after the treatment and 0 otherwise; ${\updelta }_{2}{\mathrm{CGF}}_{\mathrm{i}*}$ is the effect of the treatment on the treated firms; ${\mathrm{X}}_{\mathrm{i},\mathrm{ t}-1}$ refers to the time-varying covariates identified based on the probit regression; ${\uprho }_{\mathrm{t}}$ is a time fixed effect variable; ${\mathrm{\alpha }}_{\mathrm{i}}$ represents regional fixed effects; and ${\upvarepsilon }_{\mathrm{it}}$ is an error term. All the variables are lagged. We conduct our analysis with robust standard errors.

## Results

### Propensity score matching approach

First, a probit regression is conducted to verify the relevance of the covariates. Table 3 shows the results of this regression. The economic and financial ratios applied in the analysis, as well as the age, industry and size of the companies, are confirmed to affect the probability of participating in the CGF programme. These variables are used as time-varying covariates in the subsequent analyses.

**Table 3:** Probit regression

Dependent variable: CGF guarantee	Coefficient	Sig
Constant	0.5637 (0.0193)	0.0000***
ROE	0.0005 (0.0010)	0.0560*
ROS	0.0048 (0.0007)	0.0000***
Interest coverage ratio	− 0.0184 (0.0013)	0.0000***
Debt-to-equity ratio	− 0.0082 (0.0035)	0.0190**
Current ratio	− 0.1988 (0.0361)	0.0000***
Total assets	− 0.6521 (0.0150)	0.0000***
Age	− 0.1704	0.0000***
	(0.0006)	
North	− 0.1377 (0.0399)	0.0010**
Central	0.5296 (0.05117)	0.301
Agriculture	− 0.0703 (0.1492)	0.637
Trade	0.3914 (0.2201)	0.0750*
Services	− 0.1236 (0.1287)	0.337
Year dummies	included	
N. of obs	68,938	
Pseudo *R*^2^	0.3043	
Log likelihood	− 12,838.03	
Prob > *X*^2^	0.000	

The dependent variable is binary: it is equal to 1 for guaranteed firms and 0 otherwise. The covariates are considered at time t-1, where t is the year in which the guarantee was issued. Standard errors are in parentheses. ***, ** and * indicate significance at the 1%, 5% and 10% levels, respectively

Table 4 shows the average treatment effect on the treated firms estimated in terms of the ROI growth rate and the financial crisis indicator growth rates. The analysis is performed on the whole sample and on subsamples of micro-, small and medium enterprises. The outcome variable is the difference in the growth rates of ROI and the main business crisis indicators 1 and 2 years after the issuance of the CGF guarantees (treatment variable).

**Table 4:** Average treatment effect on the treated firms, estimated in terms of performance growth rate and financial crisis indicators growth rates: full sample and micro, small and medium firms

	Full sample	Micro firms	Small firms	Medium firms
ROI growth rate after 1 year	0.5859***	0.5394***	0.5642**	0.6248**
ROI growth rate after 2 years	0.8500***	0.8879***	0.8285**	0.6361**
Financial interest/sales growth rate after 1 year	0.3298**	0.8898***	0.1988***	0.2489***
Financial interest/sales growth rate after 2 years	0.1229*	0.8089**	0.1636***	0.2536
Debt/equity growth rate after 1 year	0.1275**	0.5512***	0.3785***	0.5321**
Debt/equity growth rate after 2 years	0.0761***	0.3168*	0.2242***	0.1083
Short-term assets/short-term liabilities growth rate after 1 year	0.2097***	0.2180***	0.0851***	0.2774*
Short-term assets/short-term liabilities growth rate after 2 years	0.2759***	0.3229***	0.1748***	0.3306***
Cash flow/total assets growth rate after 1 year	− 0.9394	− 0.4610	− 0.3519	− 0.4920
Cash flow/total assets growth rate after 2 years	0.4475	0.9342	0.2871	− 0.2680
(Tax debt + social security debt)/total assets growth rate after 1 year	− 0.4059**	− 0.0517*	− 0.8220***	− 0.8160***
(Tax debt + social security debt)/total assets growth rate after 2 years	− 0.8565***	− 0.3844*	− 0.9379***	− 0.8479*
N. of obs. 1 year	115,200	55,296	43,776	14,976
N. of obs. 2 year	98,502	48,266	37,431	13,101

This table reports the results of propensity score matching based on the k-nearest method regarding the average treatment effect on the treated firms. The results are shown for the full sample and micro, small and medium firms. The outcome variable is the difference in the growth rates of ROI and the main business crisis indicators 1 and 2 years after the issuance of the guarantee granted by the CGF (treatment variable). ***, ** and * indicate significance at the 1%, 5% and 10% levels, respectively

The guarantees help increase the difference between guaranteed and non-guaranteed firms. First, the guarantees have a strong effect on the ROI growth rate during the year after the intervention of the CGF. The impact on this variable is even greater 2 years after the issuance of the guarantee. The loans obtained due to the credit-support programme contribute, with a high level of statistical significance, to the improvement of ROI for guaranteed firms, both in the short- and medium-term. These results confirm the main findings of the accredited literature on economic additionality (Benavente et al., 2006; Caselli et al., 2019; Uesugi et al., 2010).

This effect is relevant and significant independent of the size of the focal company. Nevertheless, for medium-sized companies, the effect of a guarantee on firm profitability is slightly higher in the short-term and lower in the medium-term. Larger companies are able to exploit the loans received earlier in terms of profitability, but this impact is less significant two years after the guarantee than it is for smaller enterprises.

The consideration of further important aspects related to the ongoing concern of SMEs is the main contribution of our study. Therefore, first, our analysis reveals a relevant effect on the liquidity position of the examined guaranteed companies. The growth rate of the current ratio among the firms that have been granted public support is higher than that of companies without this support. No direct impact is recorded on the ratio of cash flow to total assets. According to our results, guarantees are not able to directly impact the ability of companies to generate cash flow. On the other hand, a significant effect is produced on the ratios related to the firms’ financial health:

- Financial interest/sales growth rate;
- Debt/equity growth rate; and
- (Tax debt + social security debt)/total assets growth rate.

The relation between interest expenses and total sales seems to worsen in the short- and medium-term. In particular, for micro-sized enterprises, obtaining a guarantee involves a significant increase in the growth rate of this ratio.

Firms that use state-backed credit incur costs. Usually, guarantees are not provided free of charge, but their costs in the focal period range from 0.25 to 1% of the guaranteed amount.

On the other hand, the interest rates of guaranteed loans should reflect the presence of a public guarantee as follows: a guaranteed loan should be more inexpensive than it would have been without the guarantee. This is also related to the inclusion of all the main public guarantee programmes on the list of credit risk mitigators of the EU Capital Requirement Regulation. Nevertheless, in addition to the guarantee fees passed onto borrowers, companies can face an increase in the interest rates applied to other unsecured loans due to the potential worsening of their financial structure and creditworthiness. Indeed, once they are accepted for public guarantees, supported companies change their financial structures and increase their debt-to-equity ratios, as shown by our main results. Growth is particularly exhibited by micro- and medium-sized enterprises in the short-term and by small firms in the medium run. There is not only an increase in the leverage of guaranteed firms compared to that of unsecured companies but also an improvement in the composition of corporate debts. The growth in a company’s “(tax debt + social security debt)/total assets” ratio decreases when it obtains a guarantee, improving its financial structure. This may be related to an increase in assets due to the new investments made possible by the guaranteed loan and/or to a partial reimbursement of old tax debts and social security debts that occurs once the new liquidity has been obtained.

Such a firm’s financial health is thus partly threatened by this new access to finance but with some positive aspects. Moreover, this deterioration in financial health is not persistent in the medium-term. The effects on the ratios “financial interest/sales” and “debt/equity” lose significance, and the coefficients decrease in the second year after the issuance of guarantees. For medium-sized companies, there is no significant relationship in the medium-term.

In summary, obtaining a guarantee helps increase the differences between guaranteed and unsecured firms, except in relation to the capacity to produce cash flow. However, while the ROI growth rate continues to grow significantly 2 years after the issuance of a guarantee, the negative effects on financial health lessen over time. Regarding the overall impact on business ongoing concerns, guarantees do not lead to an increase in business continuity risks in the medium-term. Table 5 shows the results with reference to the examined SMEs’ industries.

**Table 5:** Average treatment effect on the treated firms, estimated in terms of ROI growth rate and financial crisis indicators growth rates based on sector

	Manufacturing	Services	Trade	Agriculture
ROI growth rate after 1 year	0.7320**	0.877**	0.5136**	0.1164**
ROI growth rate after 2 years	0.7750**	0.9205***	0.8034**	0.1403*
Financial interest/sales growth rate after 1 year	0.1561*	0.6027**	0.3942**	0.2518*
Financial interest/sales growth rate after 2 years	0.1053*	0.5104*	0.3814*	0.1858*
Debt/equity growth rate after 1 year	0.2449**	0.1350**	0.9367*	0.3956
Debt/equity growth rate after 2 years	0.1340**	0.1090**	0.6887**	0.3556
Short-term assets/short-term liabilities growth rate after 1 year	0.0803*	0.2113**	0.1081*	0.0340*
Short-term assets/short-term liabilities growth rate after 2 years	0.3603**	0.2739***	0.2782*	0.1646**
Cash flow/total assets growth rate after 1 year	− 0.4985	− 0.7573	− 0.9308	− 0.7941
Cash flow/total assets growth rate after 2 years	0.1038	0.5003	0.3175	0.6762
(Tax debt + social security debt)/total assets growth rate after 1 year	− 0.2503**	− 1.5887**	− 0.4108*	− 0.4644*
(Tax debt + social security debt)/total assets growth rate after 2 years	− 0.5447**	− 1.7204**	− 1.0408**	− 0.8765**
N. of obs. 1 year	1,728	104,832	1,440	6,889
N. of obs. 2 year	1,497	89,893	1,221	5,890

The table reports the results of propensity score matching based on the k-nearest method regarding the average treatment effect on the treated firms. The results regarding the manufacturing, services, trade and agriculture industries are shown. The outcome variable is the difference in the growth rates of ROI and the main business crisis indicators 1 and 2 years after guarantees are granted by the CGF (treatment variable). ***, ** and * indicate significance at the 1%, 5% and 10% levels, respectively

The signs of the relationships are the same for every industry. Nevertheless, relevant differences in terms of magnitude are detected. In the service industry, the effects of guarantees are more evident, both in terms of profitability and in terms of crisis indicators. In particular, positive and significant increases in the growth rates of the “financial interest/sales” growth rate ratio and the “short-term assets/short-term liabilities” growth rate ratio are recorded (specifically in the short run), and a relevant decrease in the growth rate of the “(tax debt + social security debt)/total assets” ratio is highlighted. On the other hand, a higher growth rate in the debt-to-equity ratio is exhibited by the trade sector.

In general, services are usually characterized by higher long-term barriers to investment due to financial constraints. This was confirmed by a recent survey conducted by the European Investment Bank (EIB), according to which obstacles to investment activities related to the availability of finance are more significant in the services sector than other industries (EIB, 2021). This may explain the relevant impact of a guarantee for the receipt of a new loan. According to our results, the manufacturing sector did not experience a considerable impact as a result of the focal public intervention, although it is among the most capital-intensive industries (Caselli et al., 2019). This confirms, as in the case of the current ratio, that government-backed finance is aimed predominantly towards working capital and less towards investment capital during recovery phases.

Table 6 shows the main findings with reference to the type of guarantee granted by the CGF (i.e. direct guarantee or counterguarantee).

**Table 6:** Average treatment effect on the treated firms, estimated in terms of ROI growth rate and financial crisis indicators growth rate based on the type of guarantee

	Direct guarantee	Counter guarantee	Means difference
ROI growth rate after 1 year	0.2692**	0.1089**	0.1603***
ROI growth rate after 2 years	0.2984**	0.1346**	0.1638***
Financial interest/sales growth rate after 1 year	0.8984*	0.4363*	0.4621**
Financial interest/sales growth rate after 2 years	0.3564**	0.2156**	0.1408**
Debt/equity growth rate after 1 year	0.5366***	0.0329*	0.5037**
Debt/equity growth rate after 2 years	0.1152***	0.0314***	0.0838**
Short-term assets/short-term liabilities growth rate after 1 year	0.1161**	0.0939***	0.0222**
Short-term assets/short-term liabilities growth rate after 2 years	0.1933**	0.1783***	0.015**
Cash flow/total assets growth rate after 1 year	− 0.5186	− 0.7140	0.1954
Cash flow/total assets growth rate after 2 years	0.1744	0.4839	− 0.3095
(Tax debt + social security debt)/total assets growth rate after 1 year	− 0.3711***	− 0.6137***	0.2426**
(Tax debt + social security debt)/total assets growth rate 2 years	− 0.7840***	− 0.7239***	− 0.0602*
N. of obs. 1 year	62,565	52,992	
N. of obs. 2 years	53,191	45,311	

This table reports the results of propensity score matching based on the k-nearest method regarding the average treatment effect on the treated firms. The results are shown for direct guarantees and counterguarantees. The outcome variable is the difference in the ROI and main business crisis indicator growth rates 1 and 2 years after the guarantees were granted by the CGF (treatment variable). ***, ** and * indicate significance at the 1%, 5% and 10% levels, respectively. The *t*-test is used to check the equality of means of the coefficients

The results are similar for the two forms of guarantees, but as assumed, the effects have different magnitudes. Both the impact on profitability and that on the crisis indicators are more pronounced in the case of direct guarantees. The bank directly requests public guarantees mainly for companies able to achieve more substantial profit improvements in the short- and medium-term. Nevertheless, the impact of direct guarantees, on the whole, put the financial health of companies at greater risk. Higher profitability can be achieved in the face of greater negative impacts on firms’ financial structures and burdens of financial charges. However, the intervention of an MGI is able to reduce the overall risk borne by the bank and the risks to companies’ business continuity prospects simultaneously.

We conduct two diagnostic tests to verify the validity of the matching. The results are presented in Appendix 1. First, we use a *t*-test to examine differences in observable characteristics between the treatment firms and the control firms before the matching (Table 10, column 6), and we find significant differences. We calculate the descriptive statistics after the matching (Table 10, columns 1–4) and find that none of the differences in characteristics between the treated SMEs and the matched control firms are statistically significant. Second, we re-estimate the probit model restricted to the matched sample (Table 11) and observe that none of the coefficient estimates are statistically significant, suggesting that there are no distinguishable trends in the outcomes between the treated firms and the matched control firms.

### DiD methodology

The DiD methodology is applied to the whole sample and subsamples of micro, small and medium enterprises (Table 7) while considering the SMEs’ industries (Table 8) and the type of guarantee (Table 9).

**Table 7:** DiD regressions: full sample and micro-, small and medium firms

	Full sample	Micro-firms	Small firms	Medium firms
ROI growth rate after 1 year				
DiD(δ_1_)	0.4480***	0.5560***	0.320**	− 0.1253***
R^2^	0.18	0.07	0.08	0.19
ROI growth rate after 2 years				
DiD(δ_1_)	0.4577***	0.8760***	0.532***	0.0592**
R^2^	0.16	0.07	0.10	0.18
Financial interest/sales growth rate after 1 year				
DiD(δ_1_)	0.2589**	0.9232***	0.2059***	0.2685*
R^2^	0.15	0.13	0.10	0.18
Financial interest/sales growth rate after 2 years				
DiD(δ_1_)	0.1528**	0.7572***	0.1432**	0.2778
R^2^	0.14	0.10	0.11	0.16
Debt/equity growth rate after 1 year				
DiD(δ_1_)	0.1455**	0.6123***	0.3885***	0.4851**
R^2^	0.09	0.08	0.08	0.12
Debt/equity growth rate after 2 years				
DiD(δ_1_)	0.1025***	0.3325***	0.2442***	0.1854
R^2^	0.10	0.11	0.11	0.15
Short-term assets/short-term liabilities growth rate after 1 year				
DiD(δ_1_)	0.1716***	0.1998***	0.1356***	0.3258**
R^2^	0.06	0.07	0.08	0.10
Short-term assets/short-term liabilities growth rate after 2 years				
DiD(δ_1_)	0.2225***	0.3352***	0.1658***	0.4258*
R^2^	0.05	0.04	0.05	0.14
Cash flow/total assets growth rate after 1 year				
DiD(δ_1_)	− 1.1259	− 0.6598	− 0.4485	− 0.5287
R^2^	0.18	0.16	0.17	0.19
Cash flow/total assets growth rate after 2 years				
DiD(δ_1_)	− 0.5289	− 0.8526	− 0.5271	− 0.2885
R^2^	0.17	0.14	0.16	0.18
(Tax debt + social security debt)/total assets growth rate after 1 year				
DiD(δ1)	− 0.6698***	− 0.0824***	− 0.6995***	− 0.7563
R2	0.11	0.10	0.12	0.18
(Tax debt + social security debt)/total assets growth rate after 2 years				
DiD(δ1)	− 0.7225***	− 0.5992***	− 0.7556***	− 0.6587
R2	0.10	0.11	0.11	0.17

The table reports only the DiD coefficients (δ1). The outcome variable is the ROI and main business crisis indicator growth rate 1 and 2 years after the issuance of the guarantee. Regressions include controlling for time, geographical area and firm-specific variables. The results are shown for the entire sample and micro, small and medium firms. The n. of observations is approximately 119,000 (1 year) and 108,000 (2 years) for all samples; 57,000 (1 year) and 52,000 (2 years) for micro firms; 45,000 (1 year) and 39,000 (2 years) for small firms; 16,500 (1 year) and 14,700 (2 years) for medium firms. ***, ** and * indicate 1%, 5% and 10% significance levels, respectively

**Table 8:** DiD regressions: firm sector

	Manufacturing	Services	Trade	Agriculture
ROI growth rate after 1 year				
DiD(δ_1_)	0.6220**	0.6555**	0.4336**	0.1258**
R^2^	0.19	0.10	0.09	0.20
ROI growth rate after 2 years				
DiD(δ_1_)	0.7110**	1.1250**	0.8776**	0.1312*
R^2^	0.18	0.12	0.09	0.19
Financial interest/sales growth rate after 1 year				
DiD(δ_1_)	0.2051*	0.6335**	0.5556**	0.3212*
R^2^	0.17	0.09	0.08	0.18
Financial interest/sales growth rate after 2 years				
DiD(δ_1_)	0.1251*	0.6225*	0.4225*	0.3001*
R^2^	0.16	0.09	0.08	0.18
Debt/equity growth rate after 1 year				
DiD(δ_1_)	0.3675**	0.2256**	0.8856**	0.4556
R^2^	0.17	0.10	0.09	0.18
Debt/equity growth rate after 2 years				
DiD(δ_1_)	0.1156**	0.1898**	0.6995*	0.5665
R^2^	0.18	0.09	0.09	0.16
Short-term assets/short-term liabilities growth rate after 1 year				
DiD(δ_1_)	0.1656**	0.1695**	0.0985**	0.0298*
R^2^	0.08	0.09	0.07	0.10
Short-term assets/short-term liabilities growth rate after 2 years				
DiD(δ_1_)	0.2225**	0.2559**	0.2554**	0.1385**
R^2^	0.07	0.08	0.07	0.09
Cash flow/total assets growth rate after 1 year				
DiD(δ_1_)	− 0.3226	− 0.6225	− 0.6256	− 0.7865
R^2^	0.19	0.16	0.16	0.20
Cash flow/total assets growth rate after 2 years				
DiD(δ_1_)	− 0.0900	− 0.3261	− 0.3365	− 0.0256
R^2^	0.18	0.11	0.12	0.19
(Tax debt + social security debt)/total assets growth rate after 1 year				
DiD(δ1)	− 0.0566**	− 0.9859**	− 0.2699**	− 0.4458**
R2	0.17	0.16	0.17	0.19
(Tax debt + social security debt)/total assets growth rate 2 years				
DiD(δ1)	− 0.1265**	− 0.8659**	− 0.7446**	− 0.7546**
R2	0.15	0.14	0.15	0.18

This table reports only the DiD coefficients (δ1). The outcome variable is the ROI and main business crisis indicator growth rates 1 and 2 years after the issuance of the guarantees. In these regressions, we control for time, geographic area and firm-specific variables. The results are shown for the manufacturing, services, trade and agriculture industries. The n. of observations corresponding to each industry is approximately 2000 (1 year) and 1600 (2 years) for the manufacturing industry; 106,000 (1 year) and 92,000 (2 years) for the services industry; 4000 (1 year) and 3500 (2 years) for the trade industry; and 8000 (1 year) and 9000 (2 years) for the agriculture industry. ***, ** and * indicate significance at the 1%, 5% and 10% levels, respectively

**Table 9:** DiD regressions: type of guarantee

	Direct guarantee	Counter guarantee
ROI growth rate after 1 year		
DiD(δ_1_)	0.2004**	0.0988**
R^2^	0.09	0.08
ROI growth rate after 2 years		
DiD(δ_1_)	0.2668**	0.1156**
R^2^	0.08	0.07
Financial interest/sales growth rate after 1 year		
DiD(δ_1_)	0.9702*	0.5800*
R^2^	0.16	0.15
Financial interest/sales growth rate after 2 years		
DiD(δ_1_)	0.4555*	0.2665**
R^2^	0.17	0.16
Debt/equity growth rate after 1 year		
DiD(δ_1_)	0.4668**	0.0897*
R^2^	0.10	0.11
Debt/equity growth rate after 2 years		
DiD(δ_1_)	0.1336**	0.066**
R^2^	0.09	0.10
Short-term assets/short-term liabilities growth rate after 1 year		
DiD(δ_1_)	0.1222**	0.1965**
R^2^	0.08	0.07
Short-term assets/short-term liabilities growth rate after 2 years		
DiD(δ_1_)	0.2256**	0.1958**
R^2^	0.07	0.08
Cash flow/total assets growth rate after 1 year		
DiD(δ_1_)	0.6985	0.7885
R^2^	0.16	0.15
Cash flow/total assets growth rate after 2 years		
DiD(δ_1_)	0.7777	0.8695
R^2^	0.18	0.19
(Tax debt + social security debt)/total assets growth rate after 1 year		
DiD(δ1)	− 0.4589**	− 0.5106**
R2	0.06	0.07
(Tax debt + social security debt)/total assets growth rate after 2 years		
DiD(δ1)	− 0.5996**	− 0.7998**
R2	0.07	0.06

This table reports only the DiD coefficients (δ1). The outcome variable is the ROI and main business crisis indicator growth rates 1 and 2 years after the issuance of the guarantees. In these regressions, we control for time, geographic area and firm-specific variables. The results regarding guarantees and counterguarantees are shown. The n. of observations corresponding to each type is approximately 64,500 (1 year) and 55,000 (2 years) for direct guarantees and 54,000 (1 year) and 47,200 (2 years) for counterguarantees. ***, ** and * indicate significance at the 1%, 5% and 10% levels, respectively

The regression reported in Table 7 confirms the results described in Section 5.1 in terms of the ROI and financial crisis indicator growth rates of the guaranteed SMEs in the overall sample and the three subsamples based on firm size. The CGF guarantees contribute to improving the profitability of the guaranteed SMEs when compared to those without guarantees and affects their crisis indicators.

The regression shown in Table 8 confirms the results of our comparison of the ROI and financial crisis indicator growth rates of guaranteed and unsecured SMEs based on their industries. As reported in Section 5.1, the services sector exhibits the most noticeable results.

The results of the regression reported in Table 9 are consistent with those shown in Section 5.1. The effects of public guarantees on the ROI and financial crisis indicator growth rates are similar for the two types of guarantees (direct guarantees and counterguarantees), but their magnitudes differ.

**Fig. 1:**
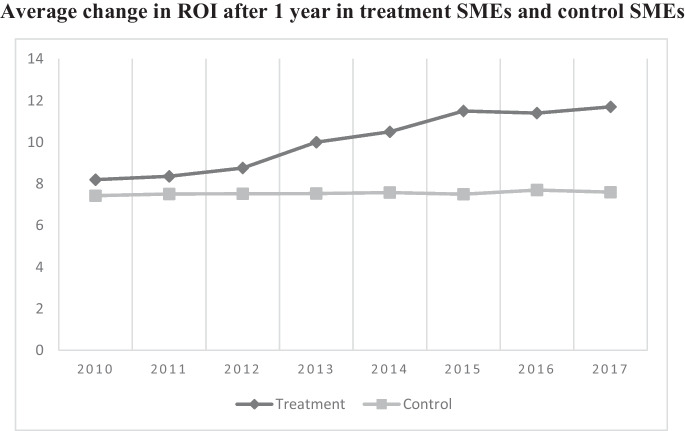
Average change in ROI after 1 year in treatment SMEs and control SMEs. *Notes.* 2010–2011 is the period before the guarantees are granted and 2012–2017 is the period after the guarantees are granted. Treatment SMEs refer to guaranteed SMEs and control SMEs refer to nonguaranteed SMEs

Following the previous literature (e.g. He & Shen, 2019; Shipilov et al, 2019), we address the validity of the DiD by considering the parallel trend assumption. If there is no treatment (i.e. the guarantee granting), the outcome variable (e.g. the change in ROI growth) of the treatment and control groups would exhibit parallel trends. Figure 1 reported in Appendix 2 shows that during the preguarantee period, the average ROI after 1 year between the guaranteed and non-guaranteed firms exhibits nearly parallel trends.^7^ This graphical evidence provides support for our use of the DiD methodology. Consistent with our results, Figure B1 also shows that during the post-guarantee period, the growth rate of the ROI after 1 year of guaranteed SMEs (i.e. treatment group) is higher than that of non-guaranteed SMEs (i.e. control group).

## Conclusions

Much existing literature highlights the benefits of public guarantee programs on subsidized companies in terms of economic performance (Lelarge et al., 2010), productivity and employment (Kang & Heshmati, 2008; Martín-García & Morán Santor, 2021), investments in intangible assets and research and development (Heshmati, 2013). On the other hand, other studies show how access to public guarantees can have negative impacts on a firm’s risk of default, especially when broad eligibility criteria are used (Lagazio et al., 2021). This paper adds to this debate by verifying the effects of public guarantees both on profitability and on crisis indicators to inform future choices about the design and implementation of guarantee programmes by public authorities and financial intermediaries.

After a severe economic crisis, governments have to decide how to revise their emergency provisions and establish new rules regarding public interventions, taking into account new information about undesired effects and emergent risks in the context of market recessions. This paper aims to support policymakers in making more effective choices, particularly in relation to designing and implementing appropriate guarantee schemes able to preserve the business continuity prospects of SMEs.

The establishment of credit support programs is characterized by many trade-offs (Anderson et al., 2021). Policymakers should take actions to optimize the balance between benefits and costs while considering all parties involved, i.e. taxpayers, SMEs and financial intermediaries. This paper attempts to draw some lessons from an analysis of the impact of public programmes during a recovery phase that are potentially applicable to the post-pandemic context.

This study shows that credit support programmes have to contend with business continuity, which is strictly connected with public funds at risk. Our results demonstrate that, in the short-term, obtaining a guarantee for the widening of credit lines deteriorates a firm’s financial equilibrium: the level and the cost of the debt of such firms increase more than those of unsecured firms. This impact on financial conditions must be adequately taken into consideration when EU state aid rules under the temporary framework are no longer in place. Indeed, under an ordinary regime, the issuance of a guarantee involves a cost, namely a percentage of the guaranteed amount. These costs will add to the general increase in interest expenses stemming from such firms’ overall debt.

Newly guaranteed loans induce a weaker financial structure among guaranteed companies in the short-term. These results confirm the advisability of imposing maximum thresholds for guarantees on individual beneficiary companies to not only control the credit risk of public funds but also avoid threatening companies’ business continuity and protecting them from the risk of debt overhang.

Moreover, when guarantees are issued, public funds should adequately assess the current level of companies’ debt and properly evaluate their ability to face increased debts in the short- and medium-term to prevent a deterioration in their financial conditions that could jeopardize their capacity to cover financial expenses. Eligible firms should be exposed to a stress test to evaluate the sustainability of their debts in the medium-term should they obtain a guarantee.

Nevertheless, our results show that public guarantees usually do not threaten firms’ ongoing concerns, given that the aforementioned deterioration of financial conditions is often accompanied by a strong improvement in firms’ economic performance, particularly among micro and small enterprises. Our main findings show that obtaining a public guarantee improves profitability in both the short- and the medium-term. Furthermore, SMEs’ financial health worsens in the short run, but burdens on financial conditions are alleviated 2 years after the issuance of the guarantee.

During recovery phases, when a “new normal” must be established, it is important to jointly evaluate financial and economic equilibria to support businesses that can achieve an effective recovery in terms of performance and financial sustainability while avoiding keeping low-productivity businesses alive.

Our main findings show that the impact of guarantees on firms’ performance and business continuity prospects depends on the selection criteria used by public schemes in terms of firm characteristics (economic sector and size) in addition to the type of intervention (direct guarantee or counterguarantee/reinsurance). Therefore, specific portions of public funds could be applied where the effects produced by such guarantees are stronger as is the case of direct guarantees granted to micro-sized enterprises and companies operating in the service sector to maximize the additionality of public resources in the context of curtailed budgets.

Currently, the CGF considers the differences among business sectors only for better assessing the probability of default among eligible firms. The kinds of ratios and thresholds used to evaluate each variable vary according to the industry in which the focal company applying for the fund operates. Considering the impact of guarantees on different sectors, to improve its economic additionality and its effects on business continuity, the CGF could consider firms’ industries also as a variable in determining the specific amounts to be allocated to establish defined economic policy objectives.

Nevertheless, during recovery phases, it is essential that public guarantee schemes do not interfere with efficient downsizing or consolidation within specific sectors to prevent dangerous zombification effects. Hence, the search for maximizing the effectiveness of public intervention must be limited to the subset of illiquid companies with viable business models in the context of targeted credit-support programmes. The use of crisis prevention indicators, which combine the analysis of economic and financial profiles, seems to be a useful reference for solving the trade-offs in the design and implementation of public guarantee funds. Our main findings thus have relevant implications for policy-makers, contributing to the public debate on rethinking credit guarantee schemes after the pandemic to find an equilibrium between effectiveness and sustainability.

Moreover, our results may have important practical implications for SMEs that are potentially eligible for a public guarantee. When applying for a public guarantee, SMEs should assess not only the impact on access to a specific financing type but should also properly estimate the medium-term effect on the overall economic and financial conditions. In this regard, our study points out that firms characterized by high levels of debt and financial distress should carefully consider their application for a public guarantee to avoid raising warning signs of a potential corporate crisis.

Despite the relevance of our results and the robust methodologies we have applied, our empirical analysis has some limitations that may be overcome by future research. First, our analysis explores the Italian context. Although this choice is useful in comparing guarantees issued in the same regulatory context and granted to eligible firms selected according to uniform criteria, a cross-country analysis could effectively verify the impact of macroeconomic and financial country-specific conditions on our main results. Such variables could help justify regulatory differences and a different level of public support for SMEs at an international level.

Second, we carried out this analysis over the period 2010–2018. It could be interesting to explore a broader period, including the most recent years, to determine if our results vary according to changed economic context. In particular, future research could test the medium-term effects of the easing of eligibility criteria and the temporary changes to the functioning of the CGF introduced during the pandemic emergency period, applying the methodology based on crisis predictive indicators presented in this paper. This is particularly important in a context of global inflation and energy crisis, where the debate on the design and implementation of support measures for SMEs is highly topical.

## Data Availability

The data that support the findings of this study are available from the corresponding author, F.I., upon reasonable request.

## Appendix 1

Table 10 (column 6) shows that before matching, there are significant differences in the covariates between the treated firms and the control group (the means difference before the matching is calculated on the value of Table 2). After matching (column 5), these differences are no longer significant. This suggests that our matching procedure is effective (i.e. the matched control group is comparable to treatment group).

**Table 10:** Differences in firm characteristics

		Guaranteed SMEs		Control sample			
	Description	(1) Mean (a)	(2) Standard deviation	(3) Mean (b)	(4) Standard deviation	(5) Means difference (a-b) After	(6) Means difference (a-b) Before
Other firm characteristics							
Firm age	N. of years	13.35	0.078	14.13	0.104	− 0.78	− 7.12***
Total assets	Log of total assets	7.48	0.0123	7.74	0.0121	− 0.26	− 2.19***
Financial data							
ROE (return on equity)	Net profit/equity	9.18	0.232	9.18	0.262	0.00	0.37***
ROS (return on sales)	EBITDA (earnings before interest, taxes, depreciation and amortization)/sales	7.39	0.165	7.39	0.211	0.00	0.54**
Interest coverage ratio	EBIT (earnings before interest and taxes)/financial interest	11.54	0.277	12.59	0.370	− 1.05	− 14.87***
Debt-to-equity ratio	Total liabilities/equity	3.06	0.312	3.07	0.363	0.01	1.25**
Current ratio	Short-term assets/short-term liabilities	0.89	0.004	0.91	0.008	− 0.02	− 0.22***

The *t*-test is used to check the equality of means. The table reports values of the main control variables after the matching and shows the diagnostic test results after and before the matching. Significance is expressed with one, two or three asterisks indicating the rejection of the null hypothesis of the coefficients with probability of 10%, 5% and 1%, respectively

Table 11 shows the probit model, restricted to the matched sample. The coefficient estimates are not statistically significant.

**Table 11:** Post-match probit regression

Dependent variable: CGF guarantee	Coefficient	Sig
Constant	0.262 (0.0183)	0.121
ROE	0.0045 (0.0013)	0.253
ROS	0.0028 (0.0008)	0.145
Interest coverage ratio	− 0.0178 (0.0011)	0.451
Debt-to-equity ratio	− 0.0079 (0.0031)	0.224
Current ratio	− 0.1883 (0.0359)	0.320
Total assets	− 0.6451 (0.0148)	0.712
Age	− 0.1604 (0.0008)	0.800
North	− 0.1737 (0.0356)	0.2401
Central	0.5396 (0.05115)	0.380
Agriculture	− 0.0630 (0.1471)	0.613
Trade	0.3691 (0.2103)	0.750
Services	− 0.1362 (0.1028)	0.331
Year dummies	Included	
Pseudo *R*^2^	0.0039	

The dependent variable is binary: it is equal to 1 for guaranteed firms and 0 otherwise. The covariates are considered at time t-1, where t is the year in which the guarantee was issued. Standard errors are in parentheses. ***, ** and * indicate significance at the 1%, 5% and 10% levels, respectively

## Appendix 2

Figure 1 shows that during the preguarantee period, the average ROI after 1 year between guaranteed and nonguaranteed firms exhibit nearly parallel trends. The figure also shows that during the postguarantee period, the growth rate of ROI after 1 year of the treatment group (the guaranteed SMEs) is higher than that of the control group (the nonguaranteed SMEs).

## Footnotes

1 The risk of “zombification” is the risk of financing nonviable companies. Unprofitable but operational firms are often referred to as “zombies”.

2 According to the EU Recommendation 2003/361, micro-enterprises have up to 9 employees and an annual turnover not exceeding € 2 million or a balance sheet total not exceeding € 2 million, while SMEs have up to 249 employees and an annual turnover not exceeding € 50 million or a balance sheet total not exceeding € 43 million.

3 A counterguarantee is a guarantee granted to an MGI and can be activated by the lender in the case of a double default (a default of the beneficiary company and the MGI).

4 Reinsurance transactions consist of the public fund’s restoration of the amount that has already been paid by the MGI to the lender within the limits of the coverage measure.

5 For more details, refer to the operating provisions of the CGF, accessible at https://www.mise.gov.it/images/stories/normativa/disposizioni_fondo_di_garanzia.pdf

6 https://www.fondidigaranzia.it, as retrieved in December 2021.

7 The same control for the other outcome variables confirms these results.

## References

[CR1] Abadie, A., Drukker, D., Herr, L. J., & Imbens, G. W. (2001). Implementing matching estimators for average treatment effects in Stata. *Stata Journal,**1*(1), 1–18. 10.1177/1536867X0400400307

[CR2] Abraham, F., & Schmukler, S. L. (2017). *Addressing the SME Finance Problem, Research and Policy Briefs from the World Bank Malaysia Hub*. Retrieved January 14, 2022, from https://documents1.worldbank.org/curated/en/809191507620842321/pdf/Addressing-the-SME-finance-problem.pdf

[CR3] AECM (2021). *SME support in the COVID crisis – The role of Guarantee Institutions*. European Association of Guarantee Institutions paper. Retrieved January 14, 2022, from https://www.smefinanceforum.org/post/sme-support-in-the-covid-crisis-the-role-of-guarantee-institutions

[CR4] Albareto, G., & Finaldi Russo, P. (2012). Financial fragility and growth prospects: Credit rationing during the crisis. Bank of Italy Occasional Paper No. 127. 10.2139/ssrn.2159210

[CR5] Altman, E. I. (1968). Financial ratio, discriminant analysis, and the prediction of corporate bankruptcy. *Journal of Finance,**23*, 589–609. 10.2307/2978933

[CR6] Anderson, J., Papadia, F., & Véron, N. (2021). COVID-19 credit-support programmes in Europe’s five largest economies. Working Paper No. 3, Bruegel. Retrieved January 14, 2022, from https://www.bruegel.org/2021/02/covid-19-credit-support-programmes-in-europes-five-largest-economies/

[CR7] Armstrong, C. E., Craig, B. R., Jackson, W. E., III, & Thomson, J. B. (2010). The importance of financial market development on the relationship between loan guarantees for SMEs and local market employment rates. Working Paper No 10–20. Federal Reserve Bank of Cleveland. 10.2139/ssrn.1723444

[CR8] Arping, S., Gyöngyi, L., & Alan, M. (2010). Public initiative to support entrepreneurs: Credit guarantees vs. co-funding. *Journal of Financial Stability,**6*(1), 26–35. 10.1016/j.jfs.2009.05.009

[CR9] Asdrubali, P., & Signore, S. (2015). The economic impact of EU guarantees on credit to SMEs–evidence from CESEE countries European Investment Fund (EIF) working paper series no. 2015/29. 10.2765/893611

[CR10] Autio, E., & Rannikko, H. (2016). Retaining winners: Van policy boost high-growth entrepreneurship? *Research Policy,**45*(1), 42–55. 10.1016/j.respol.2015.06.002

[CR11] Bartoli, F., Ferri, G., Murro, P., & Rotondi, Z. (2013). Bank-firm relations and the role of Mutual Guarantee Institutions at the peak of the crisis. *Journal of Financial Stability,**9*(1), 90–104. 10.1016/j.jfs.2012.03.003

[CR12] Beaver, W. (1966). Financial ratios as predictors of failure. Empirical research in accounting: Selected studies. *Journal of Accounting Research,**4*, 71–111. 10.2307/2490171

[CR13] Beck, T., Demirgüç-Kunt, A., Laeven, L., & Maksimovic, V. (2006). The determinants of financing obstacles. *Journal of International Money and Finance,**25*(6), 932–952. 10.1016/j.jimonfin.2006.07.005

[CR14] Beck, T., Demirgüç-Kunt, A. S. L. I., & Maksimovic, V. (2008). Financing patterns around the world: Are small firms different? *Journal of Financial Economics,**89*(3), 467–487. 10.1016/j.jfineco.2007.10.005

[CR15] Beck, T., Klapper, L. F., & Mendoza, J. C. (2010). The typology of partial credit guarantee funds around the world. *Journal of Financial Stability,**6*(1), 10–25. 10.1016/j.jfs.2008.12.003

[CR16] Benavente, J. M., Galetovic, A., & Sanhueza, R. (2006). Fogape: An economic analysis. Santiago: University of Chile Economics Department Working Paper 222. Retrieved January 14, 2022, from https://www.redegarantias.com/es/publicaciones/fogape-an-economic-analysis/

[CR17] Berger, A., & Udell, G. F. (1998). The economics of small business finance: The roles of private equity and debt markets in the financial growth cycle. *Journal of Banking and Finance,**22*(6–8), 613–673. 10.1016/S0378-4266(98)00038-7

[CR18] Berger, A., & Udell, G. F. (2006). A more complete conceptual framework for SME finance. *Journal of Banking and Finance,**30*(11), 2945–2966. 10.1016/j.jbankfin.2006.05.008

[CR19] Bertoni, F., Colombo, M. G., & Quas, A. (2018). The effects of EU-funded guarantee instruments on the performance of small and medium enterprises: Evidence from France. EIF Working Paper no. 2018/52. 10.1016/j.eap.2018.09.011

[CR20] Boocock, G., & Shariff, M. N. M. (2005). Measuring the effectiveness of credit guarantee schemes evidence from Malaysia. *International Small Business Journal,**23*(4), 427–454. 10.1177/0266242605054054

[CR21] Briozzo, A., & Cardone-Riportella, C. (2016). Spanish SMEs’ subsidized and guaranteed credit during economic crisis: A regional perspective. *Regional Studies,**50*(3), 496–512. 10.1080/00343404.2014.926318

[CR22] Bryson, A., Dorsett, R., & Purdon, S. (2002). The use of propensity score matching in the evaluation of active labour market policies. Retrieved January 14, 2022, from http://eprints.lse.ac.uk/4993/

[CR23] Calcagnini, G., Farabullini, F., & Giombini, G. (2014). The impact of guarantees on bank loan interest rates. *Applied Financial Economics,**24*(6), 397–412. 10.1080/09603107.2014.881967

[CR24] Cardone-Riportella, C., Trujillo-Ponce, A., & Briozzo, A. (2013). Analyzing the role of mutual guarantee societies on bank capital requirements for small and medium-sized enterprises. *Journal of Economic Policy Reform,**16*(2), 142–159. 10.1080/17487870.2013.801317

[CR25] Caselli, S., Corbetta, G., Rossolini, M., & Vecchi, V. (2019). Public credit guarantee schemes and SMEs profitability: Evidence from Italy. *Journal of Small Business Management,**57*(S2), 555–578. 10.1111/jsbm.12509

[CR26] Caselli, S., Corbetta, G., Cucinelli, D., & Rossolini, M. (2021). A survival analysis of public guaranteed loans: Does financial intermediary matter? *Journal of Financial Stability*, 54(C). 10.1016/j.jfs.2021.100880

[CR27] Casey, E., & O’Toole, C. M. (2014). Bank lending constraints, trade credit and alternative financing during the financial crisis: Evidence from European SMEs. *Journal of Corporate Finance,**27*, 173–193. 10.1016/j.jcorpfin.2014.05.001

[CR28] Cerulli, G. (2015). *Econometric evaluation of socio-economic programs*. Springer.

[CR29] Ciani, E., Gallo, M., Rotondi, Z. (2020). Public credit guarantees and financial additionalities across SME risk classes. Bank of Italy. Working Papers No. 1265. Retrieved January 14, 2022, from https://www.bancaditalia.it/pubblicazioni/temi-discussione/2020/2020-1265/index.html?com.dotmarketing.htmlpage.language=1

[CR30] Columba, F., Gambacorta, L., & Mistrulli, P. E. (2010). Mutual guarantee institutions and small business finance. *Journal of Financial Stability,**6*(1), 45–54. 10.1016/j.jfs.2009.12.002

[CR31] Columba, F., Gambacorta L., & Mistrulli, P.E. (2009). The effects of mutual guarantee consortia on the quality of bank lending. *Revue Bancaire et Financiere*, 4, 226–232. Retrieved January 14, 2022, from https://mpra.ub.uni-muenchen.de/17052/

[CR32] Cowan, K., Drexler, A., & Yañez, Á. (2015). The effect of credit guarantees on credit availability and delinquency rates. *Journal of Banking & Finance,**59*, 98–110. 10.1016/j.jbankfin.2015.04.024

[CR33] Cowling, M. (2010). The role of loan guarantee schemes in alleviating credit rationing in the UK. *Journal of Financial Stability,**6*(1), 36–44. 10.1016/j.jfs.2009.05.007

[CR34] Cowling, M., & Mitchell, P. (2003). Is the small firms loan guarantee scheme hazardous for banks or helpful to small business? *Small Business Economics,**21*(1), 63–71. 10.1023/A:1024408932156

[CR35] Davidsson, P., Steffens, P. R., & Fitzsimmons, J. (2008). Performance assessment in entrepreneurship research: Is there a pro-growth bias? Retrieved January 14, 2022, from http://eprints.qut.edu.au/archive/00012040

[CR36] De Jong, A., Kabir, R., & Nguyen, T. (2008). Capital structure around the world: The roles of firm- and country-specific determinants. *Journal of Banking and Finance,**32*(9), 1954–1969. 10.1016/j.jbankfin.2007.12.034

[CR37] Decramer, S., & Vanormelingen, S. (2016). The effectiveness of investment subsidies: Evidence from a regression discontinuity design. *Small Business Economics,**47*(4), 1007–1032. 10.1007/s11187.016.9749.2

[CR38] Denis, D. J., & Mihov, V. T. (2003). The choice among bank debt, non-bank private debt, and public debt: Evidence from new corporate borrowings. *Journal of Financial Economics,**70*(1), 3–28. 10.1016/S0304-405X(03)00140-5

[CR39] European Bank for Reconstruction and Development (2020). State credit guarantee schemes: Supporting SME access to finance amid the Covid-19 crisis. EBRD paper. Retrieved January 14, 2022, from https://www.ebrd.com/what-we-do/economic-research-and-data/cse-economists/state-credit-guarantee-schemes.html

[CR40] European Banking Authority, 2020. First evidence on the use of moratoria and public guarantees in the EU banking sector. EBA/Rep/2020/31. Retrieved January 14, 2022, from https://www.eba.europa.eu/sites/default/documents/files/document_library/Risk%20Analysis%20and%20Data/Risk%20Assessment%20Reports/2020/Thematic%20notes/Thematic%20note%20on%20moratoria%20and%20public%20guarantees/936761/For%20publication%20-%20Thematic%20note%20on%20moratoria%20and%20public%20guarantees.pdf

[CR41] European Commission (2020). Communication from the Commission to the European Parliament, the Coucil, the European Economic and Social Committee and the Committee of the Regions. An SME Strategy for a sustainable and digital Europe. Brussels, Com (2020), 103 final. Retrieved January 14, 2022, from https://eur-lex.europa.eu/legal-content/EN/TXT/?uri=CELEX:52020DC0103

[CR42] European Commission (2021). Annual report on European SMEs 2019/2020. European Union. Luxembourg. Retrieved January 14, 2022, from https://ec.europa.eu/growth/smes/sme-strategy/sme-performance-review_it

[CR43] European Investment Bank (2021). European Union overview; EIB Investment Survey, No. 11. Retrieved January 14, 2022, from https://www.eib.org/attachments/publications/eibis_2021_european_union_en.pdf

[CR44] European Parliament (2021). State of the SMEs Union, At a Glance, Brussels, June.

[CR45] Fazzari, S. M., Hubbard, R. G., Petersen, B. C., Blinder, A. S., & Poterba, J. M. (1988). Financing constraints and corporate investment. *Brookings Papers on Economic Activity,**1988*(1), 141–206. 10.2307/2534426

[CR46] Gai, L., Ielasi, F., & Rossolini, M. (2016). SMEs, public credit guarantees and mutual guarantee institutions. *Journal of Small Business and Enterprise Development,**23*(4), 1208–1228. 10.1108/jsbed.03.2016.0046

[CR47] Gozzi, J. C., & Schmukler, S. L. (2015). Public credit guarantees and access to finance. *European Economy: Banks, Regulation, and the Real Sector,**2*, 101–17. 10.22004/ag.econ.269324

[CR48] Graham, Y. (2004). Review of the small firms loan guarantee – interim report. HM Treasury. Retrieved January 14, 2022, from www.hmtreasury.gov.uk/graham

[CR49] He, W., & Shen, R. (2019). ISO 14001 certification and corporate technological innovation: Evidence from Chinese firms. *Journal of Business Ethics,**158*, 97–117. 10.1007/s10551-017-3712-2

[CR50] Heshmati, A. (2013). The effect of credit guarantees on SMEs’ R&D investments in Korea. *Asian Journal of Technology Innovation,**23*(3), 407–421. 10.1080/19761597.2015.1131955

[CR51] Jaffee, D. M., & Russell, T. (1976). Imperfect information, uncertainty, and credit rationing. *Quarterly Journal of Economics,**90*(4), 651–666. 10.2307/1885327

[CR52] Jorion, P., & Zhang, G. (2009). Credit contagion from counterparty risk. *Journal of Finance,**64*(5), 2053–3087. 10.1111/j.1540-6261.2009.01494.x

[CR53] Kang, J. W., & Heshmati, A. (2008). Effect of credit guarantee policy on survival and performance of SMEs in Republic of Korea. *Small Business Economics,**31*(4), 445–462. 10.1007/s11187-007-9049-y

[CR54] Kuniyoshi, S., & Tsuruta, D. (2014). Adverse selection and moral hazard in the Japanese public credit guarantee schemes for SMEs. CEPR’s Policy Portal. Retrieved January 14, 2022, from https://voxeu.org/article/adverse-selection-and-moral-hazard-japanese-credit-guarantees

[CR55] Lagazio, C., Persico, L., & Querci, F. (2021). Public guarantees to SME lending: Do broader eligibility criteria pay off? *Journal of Banking and Finance*, 133, 10.1016/j.jbankfin.2021.106287

[CR56] Lechner, M. (2002). Program heterogeneity and propensity score matching: An application to the evaluation of active labor market policies. *Review of Economics and Statistics,**84*(2), 205–220. 10.1162/003465302317411488

[CR57] Lehmann, A., & Lenaerts, K., 2021. When and how to unwind COVID support measures to the banking system? Retrieved January 14, 2022, from https://www.europarl.europa.eu/RegData/etudes/IDAN/2021/659646/IPOL_IDA(2021)659646_EN.pdf

[CR58] Leland, H. E., & Pyle, D. (1977). Informational asymmetries, financial structure and financial intermediation. *Journal of Finance,**32*(2), 371–6387. 10.1111/j.1540-6261.1977.tb03277.x

[CR59] Lelarge, C., Sraer, D., & Thesmar, D. (2010). Entrepreneurship and credit constraints: Evidence from a French loan guarantee program. In J. Lerner & A. Schoar (Eds.), *International Differences in Entrepreneurship* (pp. 243–273). University of Chicago Press.

[CR60] Leone, P., & Vento, G. A. (2012). *Credit Guarantee Institutions and SME Finance*. Palgrave Macmillan.

[CR61] Levitsky, J. (1997). Credit guarantee schemes for SMEs–An international review. *Small Enterprise Development,**8*(2), 4–17. 10.3362/0957.1329.1997.013

[CR62] Li, M. (2012). Using the propensity score method to estimate causal effects: A review and practical guide. *Organizational Research Methods,**16*(2), 1–39. 10.1177/1094428112447816

[CR63] Lin, F., Liang, D., & Chen, E. (2011). Financial ratio selection for business crisis prediction. *Experts Systems with Applications,**38*, 15094–15102. 10.1016/j.eswa.2011.05.035

[CR64] Marino, M., Lhuillery, S., Parrotta, P., & Sala, D. (2016). Additionality or crowding-out? An overall evaluation of public R&D subsidy on private R&D expenditure. *Research Policy,**45*(9), 1715–1730. 10.1016/j.respol.2016.04.009

[CR65] Martín-García, R., & Morán Santor, J. (2021). Public guarantees: A countercyclical instrument for SME growth. Evidence from the Spanish Region of Madrid. *Small Business Economics,**56*, 427–449. 10.1007/s11187-019-00214-0

[CR66] Min, S. H., Lee, J., & Han, I. (2006). Hybrid genetic algorithms and support vector machines for bankruptcy prediction. *Expert Systems with Applications,**31*(3), 652–660. 10.1016/j.eswa.2005.09.070

[CR67] Mole, K. F., Hart, M., Roper, S., & Saal, D. S. (2009). Assessing the effectiveness of business support services in England evidence from a theory-based evaluation. *International Small Business Journal,**27*(5), 557–582. 10.1177/0266242609338755

[CR68] Myers, S. (1984). The Capital Structure Puzzle. *Journal of Finance,**39*(3), 575–592. 10.1111/j.1540-6261.1984.tb03646.x

[CR69] Myers, S. C., & Majluf, N. J. (1985). Corporate financing and investment decision when firms have information that investors do not have. *Journal of Financial Economics,**13*(2), 187–221. 10.1016/0304-405X(84)90023-0

[CR70] OECD. (2020). Financing SMEs and entrepreneurs 2020: An OECD scoreboard. *OECD Publishing, Paris.*10.1787/061fe03d-en

[CR71] OECD (2011). The impact of the global crisis on SME and entrepreneurship financing and policy responses. Contribution to the OECD Strategic Response to the Financial and Economic Crisis. Retrieved January 14, 2022, from https://www.oecd.org/cfe/smes/theimpactoftheglobalcrisisonsmeandentrepreneurshipfinancingandpolicyresponses.htm

[CR72] OECD (2013). SME and entrepreneurship financing: The role of credit guarantee schemes and mutual guarantee. Retrieved January 14, 2022, from https://one.oecd.org/document/CFE/SME(2012)1/FINAL/en/pdf

[CR73] Oh, I., Lee, J. D., Heshmati, A., & Choi, G. G. (2009). Evaluation of credit guarantee policy using propensity score matching. *Small Business Economics,**33*(3), 335–351. 10.1007/s11187.008.9102.5

[CR74] Ohlson, J. A. (1980). Financial ratios and probabilistic prediction of bankruptcy. *Journal of Accounting Research,**18*(1), 109–131. 10.2307/2490395

[CR75] Ono, A., Uesugi, I., & Yasuda, Y. (2010). Examining the effects of the Emergency Credit Guarantee Program on the availability of small business credit, Institute of Economic Research Working Paper. Retrieved January 14, 2022, from https://www.ier.hit-u.ac.jp/~ifd/doc/MC2010-9s.pdf

[CR76] Panetta, I. C. (2012). An Analysis of Credit Guarantee Schemes. In P. Leone, & G. A. Vento (Eds.), *Credit Guarantee Institutions and SME Finance* (pp. 11–37). London: Palgrave Macmillan. 10.1057/9780230362321_2

[CR77] Rajan, R., & Zingales, L. (1995). What do we know about capital structure? Some evidence from international data. *J Financ,**50*, 1421–1460. 10.1111/j.1540-6261.1995.tb05184.x

[CR78] Riding, A., Madill, J., & Haines, G. (2007). Incrementality of SME loan guarantees. *Small Business Economics,**29*(1–2), 47–61. 10.1007/s11187.005.4411.4

[CR79] Rostamkalaei, A., & Freel, M. (2016). The cost of growth: Small firms and the pricing of bank loans. *Small Business Economics,**46*(2), 255–272. 10.1007/s11187.015.9681.x

[CR80] Rubin, D. B. (2004). Direct and Indirect Causal Effects via Potential Outcomes. *Scandinavian Journal of Statistics,**31*(2), 161–170. 10.1111/j.1467-9469.2004.02-123.x

[CR81] Saito, K., & Tsuruta, D. (2014). Information asymmetry in SME credit guarantee schemes: Evidence from Japan. Discussion Paper Series 14-E-042. Retrieved January 14, 2022, from https://www.rieti.go.jp/jp/publications/dp/14e042.pdf

[CR82] Samujh, R. H., Twiname, L., & Reutemann, J. (2012). Credit guarantee schemes supporting small enterprise development: A review. *Asian Journal of Business and Accounting*, 5(2), 21–40. Retrieved January 14, 2022, from https://researchcommons.waikato.ac.nz/bitstream/handle/10289/7079/AJBA%205(2)%20Samujh,%20Twiname%20and%20Reutemann%202012.pdf?sequence=1

[CR83] Schmidt, A., & Van Elkan, M. (2010). *Quantification of the Macroeconomic Effects of the Activities of German Guarantee Banks under the Framework Conditions of the Global Financial and Economic Crisis*. Inmit, University of Trier, Germany. Retrieved January 14, 2022, from https://aecm.eu/wp-content/uploads/2015/07/inmit-study-on-the-macroeconomic-benefits-of-the-german-guarantee-banks1.pdf

[CR84] Shipilov, A. V., Greve, H. R., & Rowley, T. J. (2019). Is all publicity good publicity? The impact of direct and indirect media pressure on the adoption of governance practices. *Strategic Management Journal,**40*, 1368–1393. 10.1002/smj.3030

[CR85] Stiglitz, J., & Weiss, A. (1981). Credit rationing in markets with imperfect information. *American Economic Review,**71*(2), 393–409. 10.7916/D8V12FT1

[CR86] Uesugi, I., Sakai, K., & Yamashiro, G. M. (2010). The effectiveness of public credit guarantees in the Japanese loan market. *Journal of the Japanese and International Economies,**24*(4), 457–480. 10.1016/j.jjie.2010.08.001

[CR87] Ughetto, E., Scellato, G., & Cowling, M. (2017). Cost of capital and public loan guarantees to small firms. *Small Business Economics,**49*(2), 1–19. 10.1007/s11187.017.9845.y

[CR88] Van der Wijst, N., & Thurik, R. (1993). Determinants of small firm debt ratios: An analysis of retail panel data. *Small Business Economics,**5*(1), 55–65. 10.1007/BF01539318

[CR89] Vogel, R. C., & Adams, D. W. (1997). The benefits and costs of loan guarantee programs. *The Financier,**4*(1), 22–29. 10.4236/jtts.2019.92008

[CR90] Zecchini, S., & Ventura, M. (2009). The impact of public guarantees on credit to SMEs. *Small Business Economics,**32*(2), 191–206. 10.1007/s11187.007.9077.7

